# Cardiotoxicity related to intrapericardial infusion of bevacizumab in the treatment of lung cancer-mediated malignant pericardial effusion: a case report

**DOI:** 10.3389/fphar.2025.1573297

**Published:** 2025-10-17

**Authors:** Shilong Wu, Yanjun Lu, Chenyang Xu, Huafeng Liu

**Affiliations:** ^1^ Department of Thoracic Surgery, Ganzhou People’s Hospital, Ganzhou, China; ^2^ Department of Oncology, Ganzhou People’s Hospital, Ganzhou, China

**Keywords:** bevacizumab, cardiotoxicity, myocardial ischemia, lung cancer, malignant pericardial effusion

## Abstract

**Background:**

Lung cancer can result in malignant pericardial effusion (MPE), impacting patient prognosis. Intrapericardial infusion of bevacizumab was an alternative treatment for MPE.

**Case presentation:**

We present the case of a 48-year-old female with stage IV lung adenocarcinoma and MPE. MPE was managed by intrapericardial infusion of bevacizumab. The first intrapericardial infusion of bevacizumab effectively controlled the MPE for 8 months. Cardiotoxicity quickly emerged after the second intrapericardial infusion of bevacizumab. After intensive treatment, the symptoms of cardiotoxicity resolved within 10 days.

**Conclusion:**

The present case indicates that intrapericardial infusion of bevacizumab could lead to cardiotoxicity in MPE patient. Cardiac examinations should be conducted before and after anti-vascular endothelial growth factor treatment.

## Introduction

Malignant pericardial effusion (MPE) commonly develops in cancer patients, leading to refractory cardiac dysfunction and pericardial tamponade (Chahine et al., 2021). MPE is linked to high recurrence rates and a poor prognosis (Gornik et al., 2005; Dequanter et al., 2008). Commom clinical treatments for MPE include pericardiocentesis, percutaneous catheter drainage, pericardial window, systemic antineoplastic treatment and intrapericardial infusion of therapeutic agents (Zhang et al., 2020). Intrapericardial infusion of bevacizumab was an alternative treatment for MPE due to its effectiveness and safety (Chen et al., 2015; Chen et al., 2016; Ueda et al., 2016; Del Rosario et al., 2016). Here, we reported a MPE patient who experienced cardiotoxicity after intrapericardial infusion of bevacizumab.

## Case report

**FIGURE 1:**
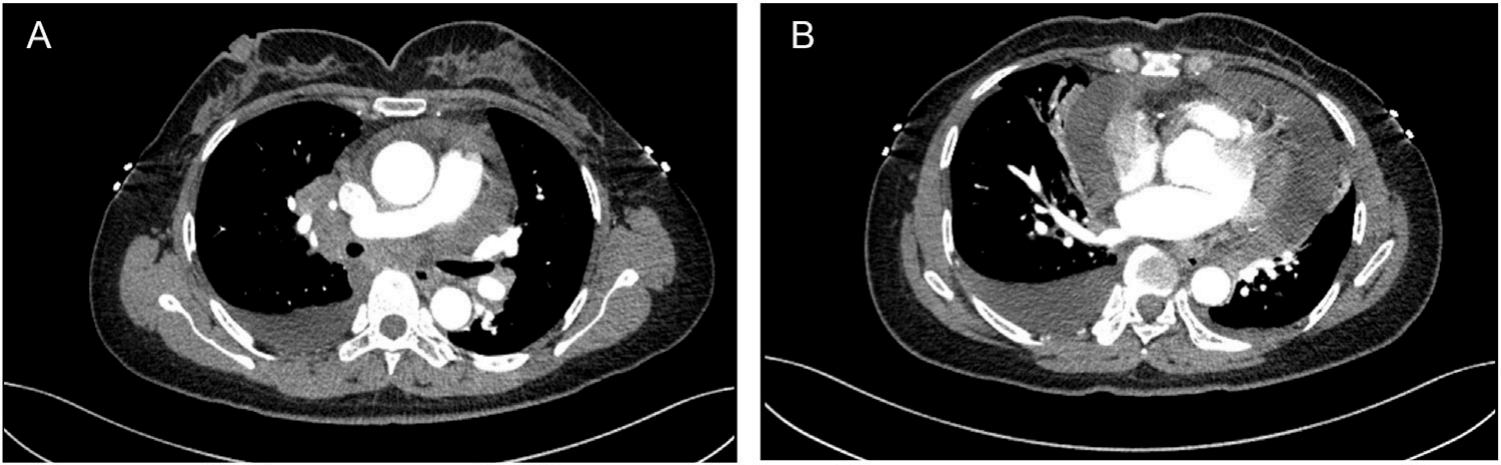
Chest computed tomography revealed a right hilar mass **(A)**, pleural and pericardial effusion **(B)**.

**FIGURE 2:**
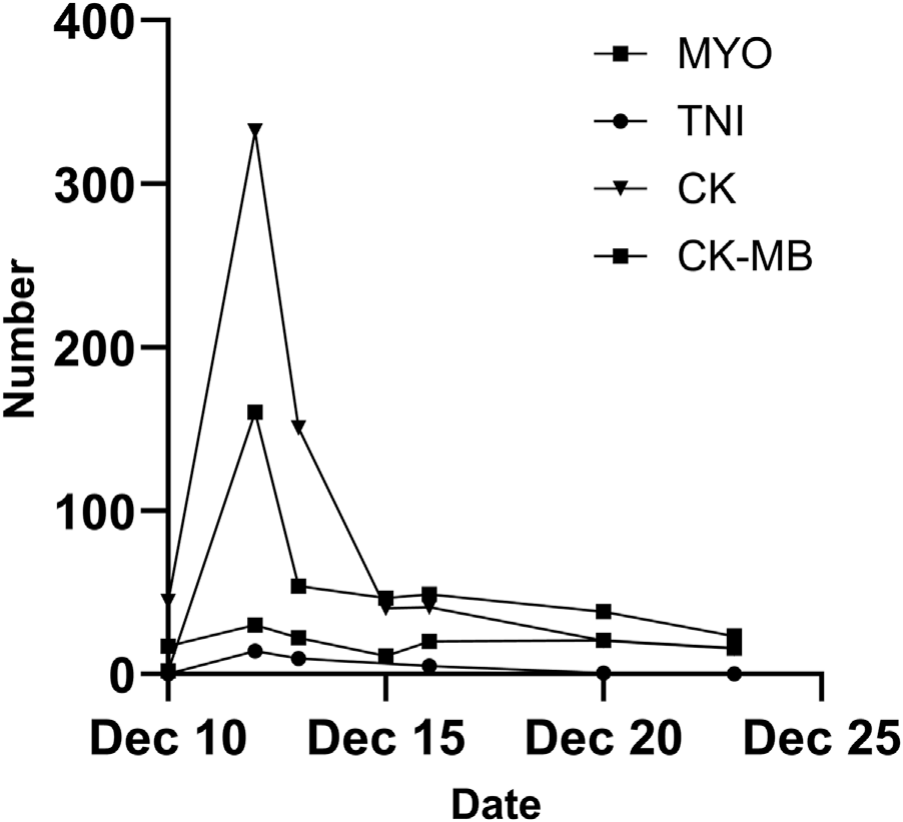
Alterations in myocardial enzyme levels during treatment. MYO, myoglobin; TNI, troponin I; CK, creatine kinase; CK-MB, creatine kinase isoenzyme MB.

**FIGURE 3:**
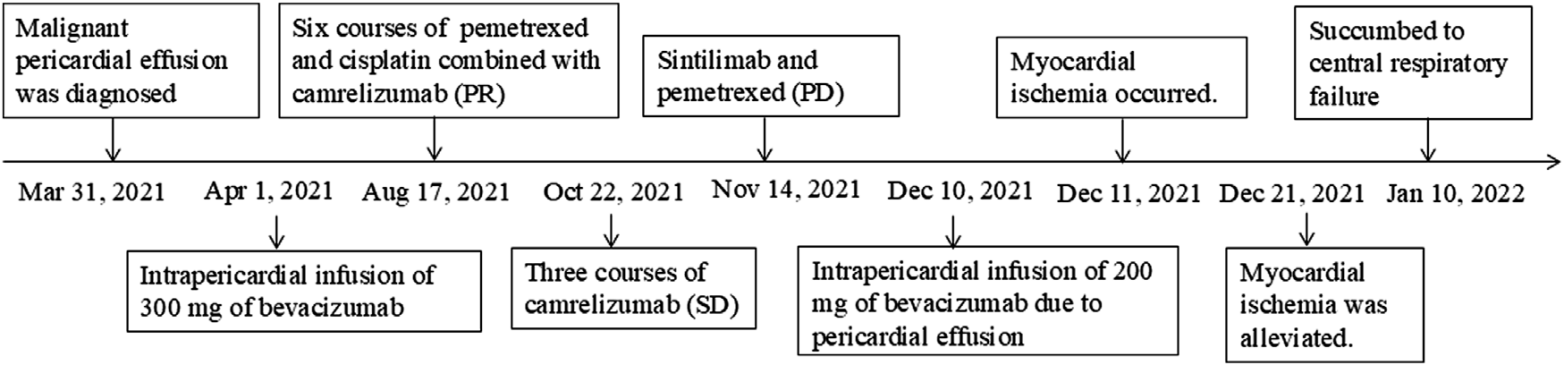
Schematic of the patient’s treatment history.

A 48-year-old female was admitted to a local hospital because of tussiculation and chest tightness on 23 March 2013. Chest computed tomography revealed a right hilar mass (Figure 1A), pleural effusion (Figure 1B), and pericardial effusion (Figure 1B). Right thoracic puncture and drainage were performed, and pleural effusion examination found adenocarcinoma cells. Needle aspiration cytology of the right supraclavicular lymph node indicated adenocarcinoma. The patient came to our center for further treatment on 31 March 2021. Cervical lymph node biopsy indicated metastatic adenocarcinoma which came from lung. Pericardiocentesis and drainage were performed, and pericardial effusion examination found adenocarcinoma cells. The patient was diagnosed with right lung adenocarcinoma with pleural and pericardial metastases (cT1N3M1a). Next-generation sequencing showed no driver gene mutations. On 1 April 2021, the patient received the first intrapericardial infusion of bevacizumab (300 mg). From April 8 to 17 August 2021, she completed six cycles of first-line therapy with pemetrexed and cisplatin combined with camrelizumab. This was followed by three cycles of camrelizumab maintenance therapy from September 9 to 22 October 2021. Due to disease progression, treatment was switched to sintilimab plus pemetrexed on 14 November 2021. Throughout this period, electrocardiograms and myocardial enzyme levels remained within normal limits, and the pericardial effusion was well-controlled, with only a minimal amount remaining. On 10 December 2021, the patients received the second intrapericardial infusion of 200 mg of bevacizumab due to worsening pericardial effusion. The following day (December 11), the patient developed acute chest pain. Myocardial enzymes (Figure 2) were significantly elevated and electrocardiography (Supplementary Data Sheet S1) showed ST-T changes and QTc prolongation. The N-terminal pro-brain natriuretic peptide level was 11,507 pg/mL and echocardiogram showed a normal ejection fraction. Bevacizumab-associated cardiotoxicity (myocardial ischemia) was initially suspected. Methylprednisolone, aspirin, and heparin were administered for treatment. The myocardial enzyme levels trended downward within 2 days, and the chest pain resolved after 10 days. Unfortunately, the patient died of central respiratory failure on 10 January 2022. Figure 3 presented the patient’s treatment timeline.

## Discussion

Malignant tumors are a common cause of pericardial effusion, with lung cancer being the most prevalent. 9 Some studies have shown that intrapericardial infusion of bevacizumab is a safe and effective treatment for managing MPE (Chen et al., 2015; Chen et al., 2016; Chen et al., 2017; He et al., 2022). Bevacizumab seldom leads to cardiotoxicity, a potentially life-threatening side effect (Abdel-Qadir et al., 2017). To the best of our knowledge, this is the first report on a MPE patient who underwent myocardial ischemia possibly due to intrapericardial infusion of bevacizumab.

There is currently no standard effective treatment for MPE. The treatment of MPE focuses on alleviating symptoms, ensuring hemodynamic stability, and preventing fluid recurrence. Systemic chemotherapy is effective for MPE and intracardiac drug injection benefits patients without recurrence (Zhang et al., 2020). Bevacizumab is a recombinant humanized monoclonal antibody that inhibits the binding of human vascular endothelial growth factor (VEGF) to its receptors (Presta et al., 1997). It has been shown to inhibit the growth of 13 types of malignant cells and reduce the density, diameter, and permeability of blood vessels (Takahashi et al., 1997). Bevacizumab is typically given intravenously, but its intracavitary administration is a safe and effective method for managing MPE (Chen et al., 2015; Chen et al., 2016; Chen et al., 2017; He et al., 2022). A randomized clinical study demonstrated that intrapleural infusion of bevacizumab is more effective and safer than intravenous infusion for managing malignant pleural effusion due to lung cancer (Nie et al., 2020). Compared to chemotherapy drugs like platinum alone, intracardial injection of bevacizumab decreased side effects and had favorable outcomes in patients with MPE (Chen et al., 2016; Chen et al., 2017; He et al., 2022). The most common adverse events associated with bevacizumab were hypertension, proteinuria, asthenia, and diarrhea (Geiger-Gritsch et al., 2010). A meta-analysis revealed that VEGF signaling pathway inhibitors significantly raise the odds of hypertension, cardiac ischemia, arterial thromboembolism, and cardiac dysfunction by 5.3, 2.8, 1.5, and 1.4 times, respectively. The risk of a fatal cardiovascular event with VEGF inhibitor therapy is only 0.25% (Abdel-Qadir et al., 2017). Diagnosis and treatment of chronic and acute coronary syndromes following VEGF inhibitor therapy adhere to ESC Guidelines on cardio-oncology (Lyon et al., 2022). The patient developed myocardial ischemia symptoms 1 day after a pericardial infusion of bevacizumab, having undergone multiple previous antineoplastic treatments without cardiac toxicity. The symptoms of myocardial ischemia subsided after treatment, but the ECG revealed a prolonged QTc interval. QTc interval prolongation is linked to the activation of cardiomyocyte potassium channel proteins and can be induced by VEGF inhibitors (Mihalcea et al., 2023). Management and treatment of cardiac ischemia induced by VEGF inhibitors should be tailored to the patient’s cancer severity, life expectancy, comorbidities, and available highly effective antineoplastic alternatives (Lyon et al., 2022). Bevacizumab carries a theoretical risk of inducing myocardial ischemia through mechanisms such as inhibition of angiogenesis, impairment of coronary microcirculation, or induction of localized vasospasm (Mihalcea et al., 2023). Although the onset of myocardial ischemic symptoms coincided temporally with the second intracardiac administration of bevacizumab, the potential cumulative cardiotoxic effects of prior treatments—including cisplatin, camrelizumab, and sintilimab—cannot be ruled out as contributing factors (Senkus and Jassem, 2011). The existing literature primarily highlights its efficacy and short-term tolerability, but robust data from large, prospective trials are lacking. Therefore, the occurrence of a serious adverse event like myocardial ischemia in our patient underscores the potential risks and the necessity for heightened vigilance.

In conclusion, the present case indicates that intrapericardial infusion of bevacizumab could lead to cardiotoxicity in MPE patient. Intrapericardial infusion of bevacizumab is a safe and effective treatment for managing MPE, but monitoring for adverse effects is essential. The efficacy of bevacizumab in treating cancer must be weighed against its potential cardiotoxicity.

## Data Availability

The original contributions presented in the study are included in the article/Supplementary Material, further inquiries can be directed to the corresponding author.

## Abbreviations

MPE, Malignant pericardial effusion; VEGF, human vascular endothelial growth factor.
