# Supplementary material for: Cardiotoxicity related to intrapericardial infusion of bevacizumab in the treatment of lung cancer-mediated malignant pericardial effusion: a case report
Source: Front Pharmacol. 2025 Oct 17;16:1573297. doi: 10.3389/fphar.2025.1573297 (PMC12575337; doi:10.3389/fphar.2025.1573297)
Supplement: Supplementary file 1 [file DataSheet1.pdf]

赣州市人民医院心电图报告单

)　　　　日期:2021/11/9 9:00:44

P:102ms　　QT/QTc:330/413ms　　心　率:94 bpm
QRS:82ms　　QRS电轴:+21°
P-R:152ms　　RV5/SV1:1.08/0.55mV

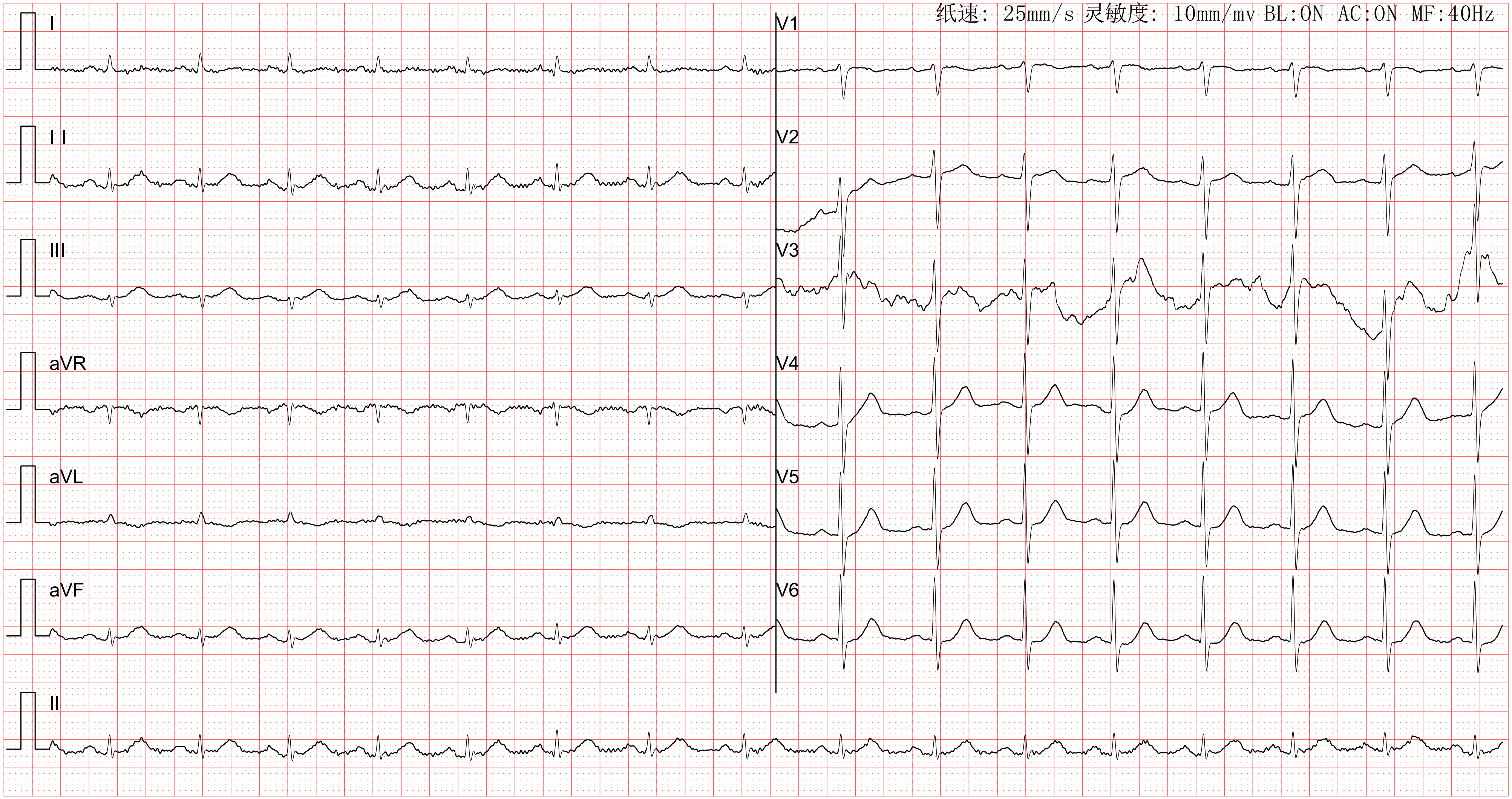

诊断提示:

1、窦性心律
2、肢体导联低电压

赣州市人民医院心电图报告单

)

日期:2021-11-30 15:11:15

P:88ms
QRS:88ms
P-R:150ms

QT/QTc:378/460ms
QRS电轴:0°
RV5/SV1:0.73/0.54mV

心　率:89 bpm

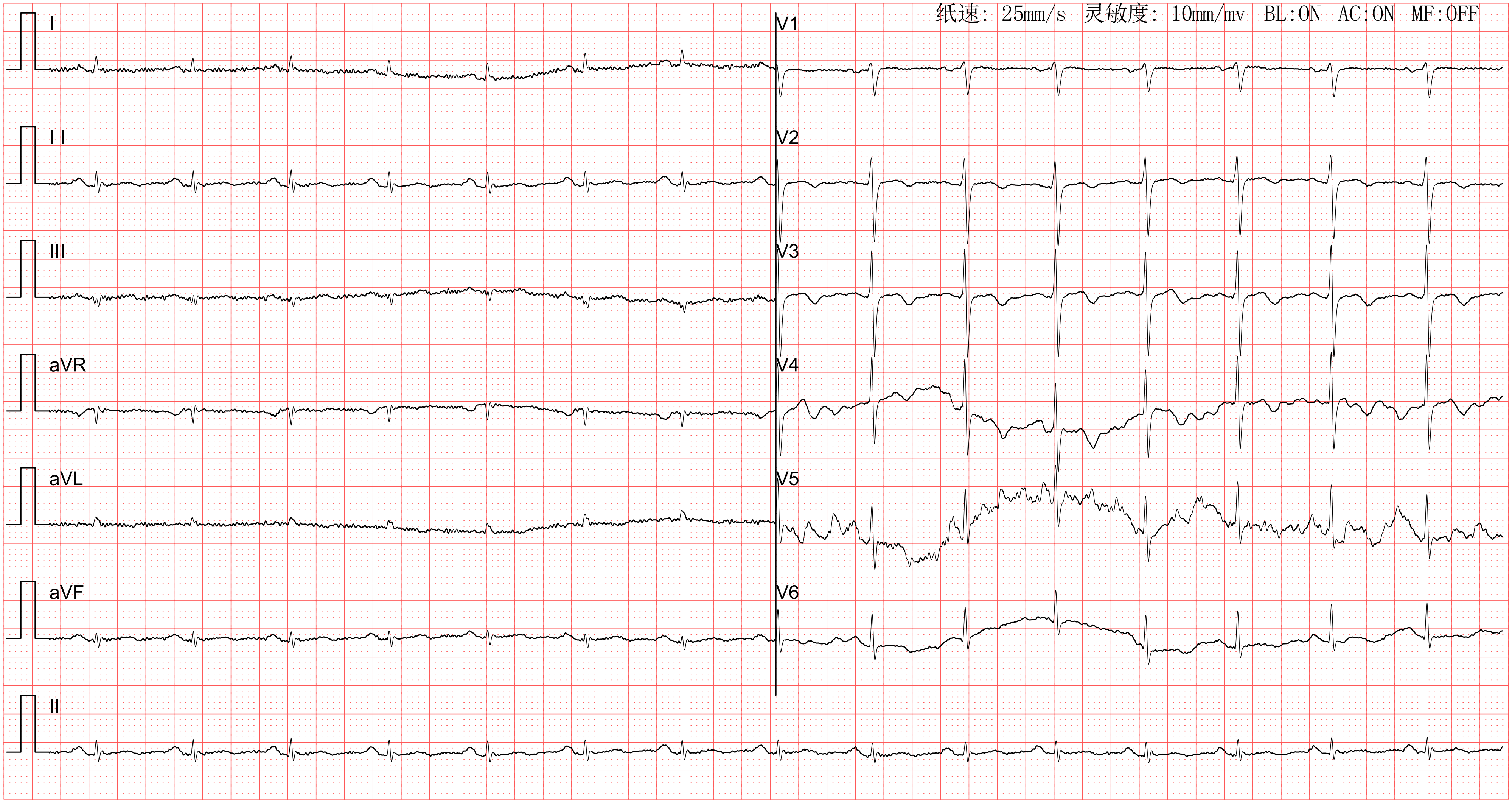

诊断提示:

1、窦性心律
2、肢体导联低电压

赣州市人民医院心电图报告单

日期:2021/12/11 11:13:50

P:96ms QT/QTc:410/518ms 心 率:96 bpm
QRS:86ms QRS电轴:+28°
P-R:154ms RV5/SV1:1.14/0.93mV

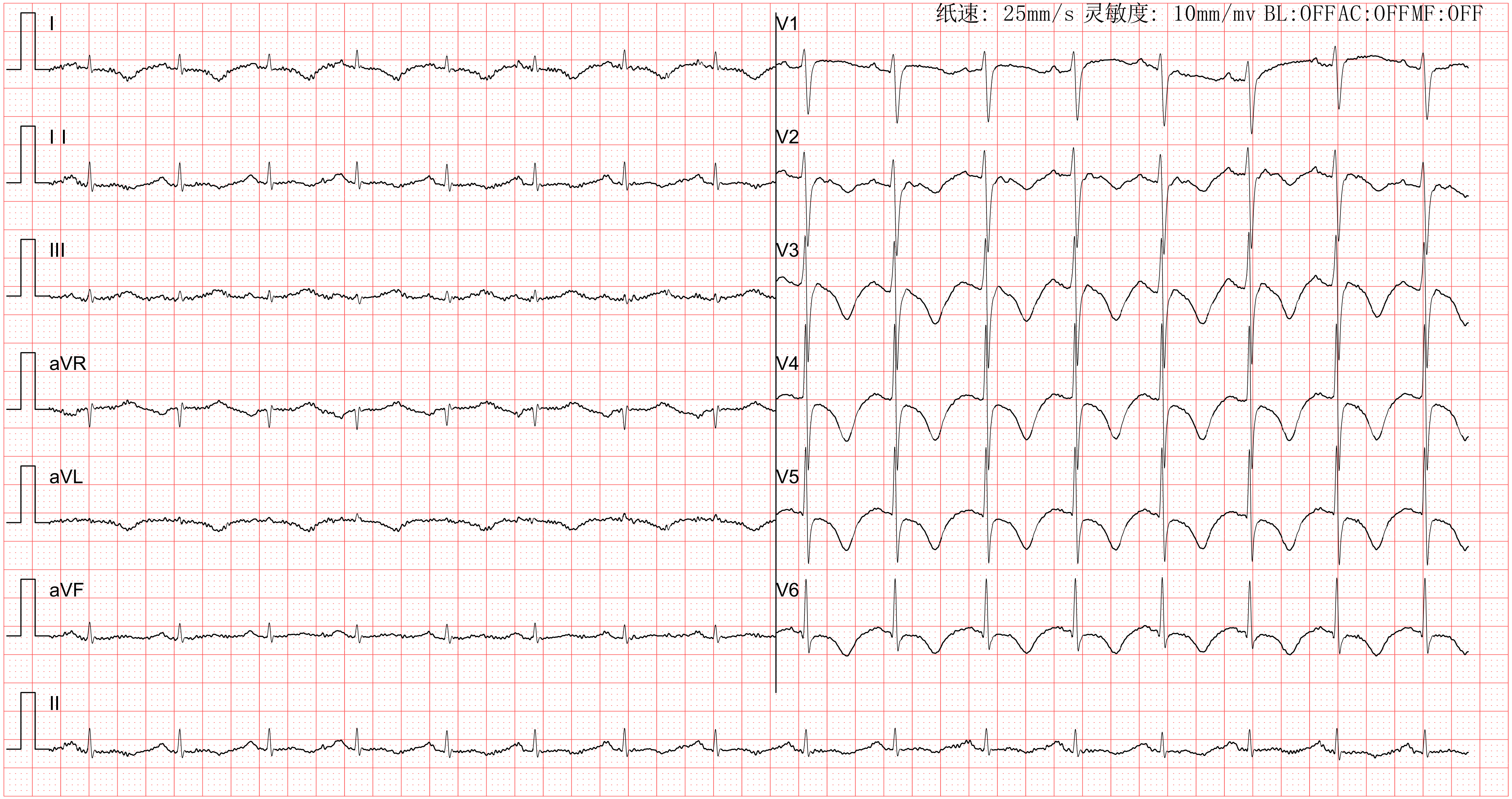

诊断提示:

1、窦性心律
2、ST-T改变，请结合临床
3、QTc间期延长延长，建议监测电解质，密切观察
4、肢体导联低电压

赣州市人民医院赣州市人民医院心电图报告单

日期:2021-12-12 09:32:07

P:76ms
QRS:80ms
P-R:120ms

QT/QTc:384/490ms
QRS电轴:+74°
RV5/SV1:0.95/1.08mV

心　率:98 bpm

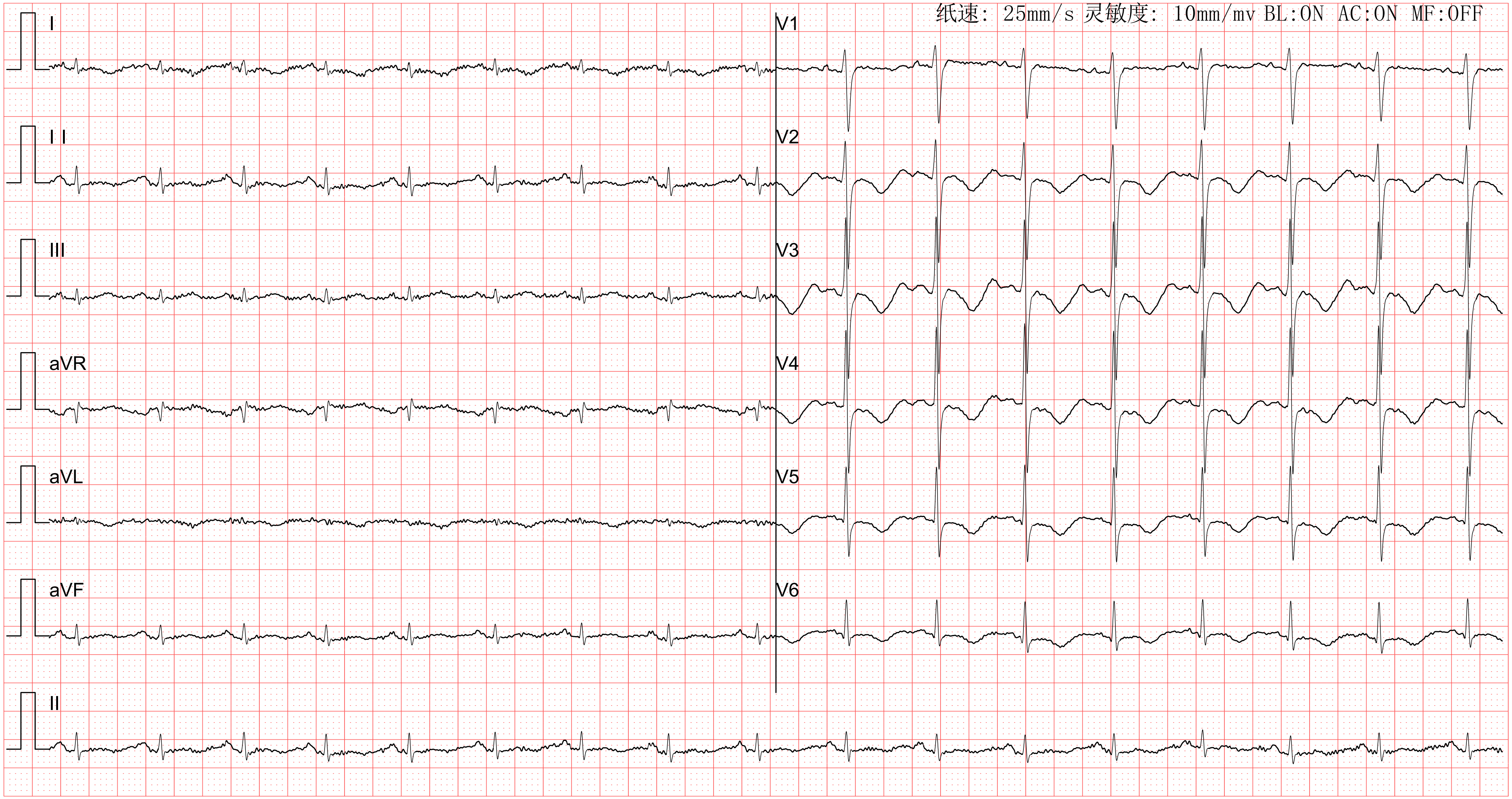

诊断提示:

1、窦性心律
2、肢体导联QRS低电压
3、ST-T改变，请结合临床。
4、QTC间期延长

赣州人民医院心电图报告单

日期:2021-12-17 11:11:02

P:96ms
QRS:90ms
P-R:124ms

QT/QTc:386/467ms
QRS电轴:+14°
RV5/SV1:1.20/0.66mV

心　率:88 bpm

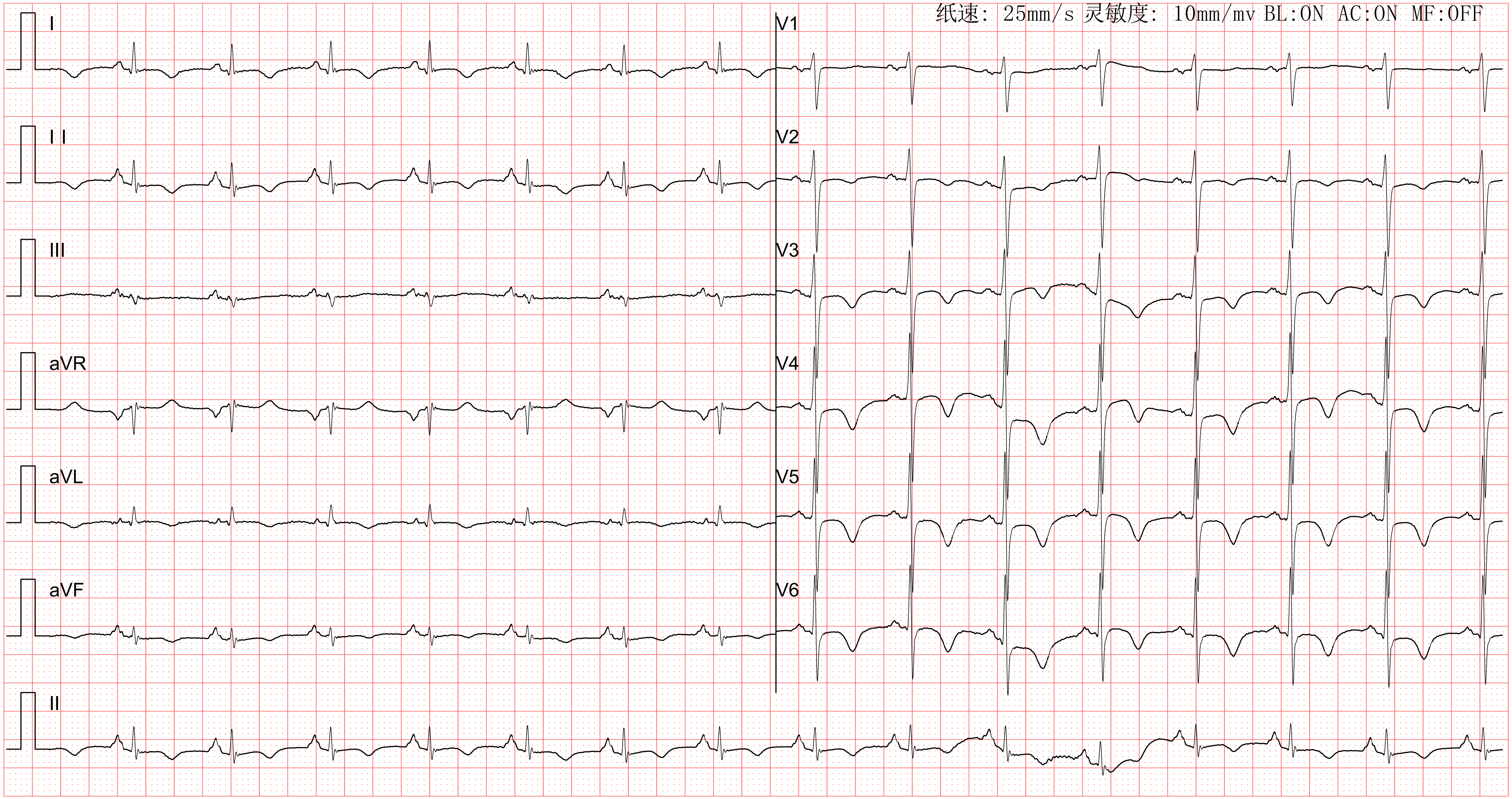

诊断提示:

1、窦性心律
2、ST-T改变
3、QTc间期延长
